# Experimental multiparametric magnetic resonance imaging characterization of iliocaval venous thrombosis pathological changes

**DOI:** 10.1016/j.jvsv.2024.101895

**Published:** 2024-04-26

**Authors:** Louis Magnus, Adeline Schwein, Ponraj Chinnadurai, Killian Fontaine, Kyle Autry, Dipan J. Shah, Kathryn Jane Grande-Allen, Nabil Chakfé, Jean Bismuth

**Affiliations:** a Department of Vascular and Endovascular Surgery, Gabriel Montpied Hospital, University Hospital of Clermont-Ferrand, Clermont-Ferrand, France; b Department of Vascular and Endovascular Surgery, Sir Charles Gairdner Hospital, Perth, Western Australia, Australia; c Heart and Vascular Research Institute, Harry Perkins Medical Research Institute, Perth, Western Australia, Australia; d Occam Labs, Santa Cruz, CA; e Houston Methodist DeBakey Heart & Vascular Center, Houston Methodist Hospital, Houston, TX; f Department of Bioengineering, BioScience Research Collaborative, Rice University, Houston, TX; g Department of Vascular Surgery, Kidney Transplantation and Innovation, University Hospital of Strasbourg, Strasbourg, France; h GEPROMED, Strasbourg, France; i Division of Vascular Surgery, USF Health Morsani School of Medicine, Tampa, FL

**Keywords:** Animal model, Iliocaval obstruction, Magnetic resonance imaging, Venous thrombosis

## Abstract

**Objective:**

Iliocaval thrombotic obstruction is a challenging condition, especially because thrombus age and corresponding pathological remodeling at presentation are unknown, which directly impacts management. Our aim was to assess the ability of magnetic resonance imaging (MRI) in determining age thresholds of experimentally created inferior vena cava (IVC) thrombosis in pigs.

**Methods:**

We used a previously described swine model of IVC thrombosis. The animals underwent MRI at baseline, immediately after thrombosis creation, and after a follow-up period extending from 2 to 28 days. Thirteen pigs were divided into three groups according to disease chronicity: acute group (AG; n = 5), subacute group (SAG; n = 4), and chronic group (CG; n = 4), with a mean thrombosis age of 6.4 ± 2.5 days, 15.7 ± 2.8 days, and 28 ± 5.7 days, respectively. A T_1_-weighted volumetric interpolated breath-hold examination sequence was used to anatomically delineate IVC thrombus as a region of interest. Three other MRI sequences were used to assess the thrombus signal.

**Results:**

The Kruskal-Wallis test showed a statistically significant difference in T_1_ relaxation times after contrast injection (*P* = .026) between the three groups of chronicity. The AG (360.2 ± 102.5 ms) was significantly different from the CG (336.7 ± 55.2 ms; *P* = .003), and the SAG (354.1 ± 89.7 ms) was significantly different from the AG (*P* = .027). There was a statistically significant difference in native T_2_ relaxation times (*P* = .038) between the three groups. The AG (160 ± 86.7 ms) was significantly different from the SAG (142.3 ± 55.4 ms; *P* = .027), and the SAG was significantly different from the CG (178.4 ± 11.7 ms; *P* = .004).

**Conclusions:**

This study highlighted MRI characteristics in a swine model that might have the potential to significantly differentiate subacute and chronic stages from an acute stage of deep vein thrombosis in humans. Further clinical studies in humans are warranted.

**Clinical Relevance:**

In addition to providing a better understanding of venous thrombosis remodeling over time, magnetic resonance imaging has the potential to be a tool that could allow us to characterize the composition of venous thrombus over an interval, allowing for a refined analysis of the local evolution of venous thrombosis. We propose a noninvasive and innovative method to characterize different thresholds of chronicity with magnetic resonance imaging features of central deep vein thrombosis of the inferior vena cava experimentally obtained using a totally endovascular in vivo swine model, mimicking human pathophysiology. Being able to determine these features noninvasively is critical for vascular specialists when it comes to choosing between fibrinolytic therapy, percutaneous thrombectomy, or surgical management.

**Article Highlights**

• **Type of Research:** An experimental, prospective, longitudinal, observational study.
• **Key Findings:** We propose a noninvasive and innovative method to characterize different thresholds of chronicity with magnetic resonance imaging features of central deep vein thrombosis of the inferior vena cava experimentally obtained using a totally endovascular in vivo swine model, mimicking human pathophysiology.
• **Take Home Message:** In addition to providing a better understanding of venous thrombosis remodeling over time, magnetic resonance imaging has the potential to be a tool that could allow us to characterize the composition of venous thrombus over an interval, allowing for a refined analysis of the local evolution of venous thrombosis.

Iliocaval obstruction is a significant cause of morbidity in patients with a spectrum of symptoms, including pulmonary embolus, painful lower extremity swelling, threatened limb, and recurrent deep vein thrombosis (DVT).^1^ The etiology of venous thrombosis is multifactorial, involving risk factors such as genetics, venous endothelial injury, hypercoagulability, and venous stasis.^2^

One of the unknown factors is the ideal timing of treatment after symptom onset. The success of venous recanalization, preservation of valve function, and symptom relief might depend on the timing of treatment. A delay to symptom onset and clinical history are commonly used to determine thrombus age, with, however, a certain subjectivity and lack of reliability.^3^ Moreover, thrombus occurs before symptoms onset, especially in the event of partial occlusion of the vein. Acute thrombi are thought to be easier to mechanically retrieve and to be more responsive to thrombolysis. The 14-day threshold has been shown to be a factor associated with better results of thrombus removal strategies.^4^ However, the threshold lacks precision, because it is necessary to base it on the duration of the patient's symptoms to estimate this period.

Apart from ultrasound, which can routinely distinguish with some accuracy the chronicity of DVT,^5^ noninvasive modalities are lacking to precisely assess the actual age or stage of the thrombus and venous wall properties, which greatly limits targeted treatment. A few studies have assessed the ability of identifying thrombus content using noninvasive imaging modalities, especially with magnetic resonance imaging (MRI). Andia et al^6^ showed that the use of a fibrin-specific contrast agent could accurately estimate thrombus fibrin content in a mouse model and, thus, identify thrombi amenable for thrombolysis. Two other studies were reported by the same team highlighting specific MRI sequences that can be used for staging of thrombus composition in a mouse model.^7^^,^^8^

The objective of our study was to assess the ability of multiparametric MRI in determining age thresholds for experimentally created inferior vena cava (IVC) thrombosis in pigs. Ultimately, the goal was to define MRI as a multiparametric imaging modality that would allow noninvasive assessment of specific stages of venous remodeling.

## Methods

The in vivo experiments reported in this study were performed in accordance with the ARRIVE (Animal Research: Reporting of In Vivo Experiments) guidelines.^9^ The experiments were done in strict accordance with the Guide for the Care and Use of Laboratory Animals of the National Institutes of Health. Our institutional animal care and use committee approved the present study (protocol No. AUP-0615-0049).

### Animal population

**Fig 1:**
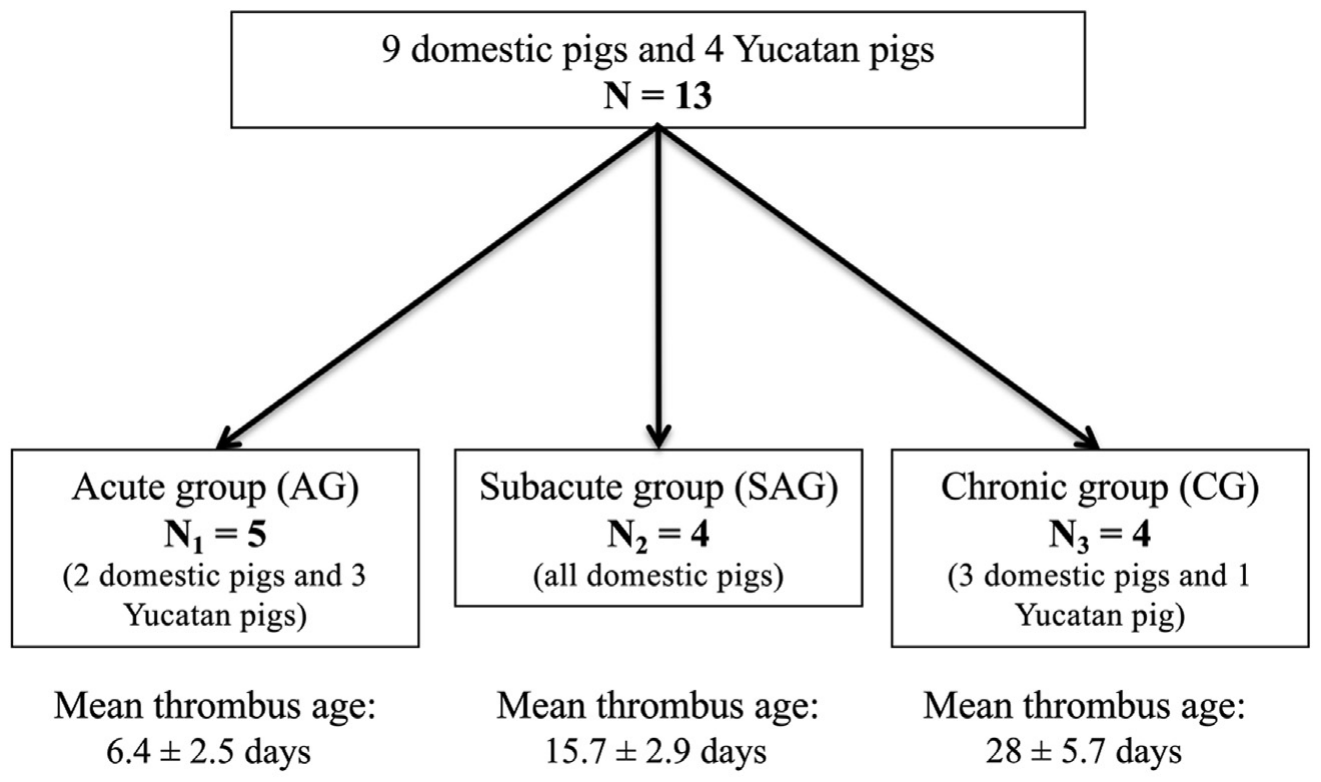
Flow chart presenting the distribution of animals into three groups according to the chronicity of the disease.

Between February 2016 and February 2018, we successfully created IVC thrombosis in 13 female pigs in a dedicated research hybrid operating room at the Methodist Institute for Technology, Innovation and Education. The 13 pigs included 9 domestic pigs (USDA class A; Oak Hill Genetics) with a mean weight of 57 ± 5.2 kg, and 4 Yucatan pigs (USDA class A; S & S Farms), with a mean weight of 61.9 ± 5.8 kg. These 13 pigs were divided into three groups according to the chronicity of the IVC thrombosis created (Fig 1).

### Experimental protocol

All the pigs were maintained under general anesthesia and endotracheal mechanical ventilation for the duration of the procedures, under strict veterinary supervision in accordance with the Houston Methodist Hospital institutional animal care and use committee protocols.

**Fig 2:**
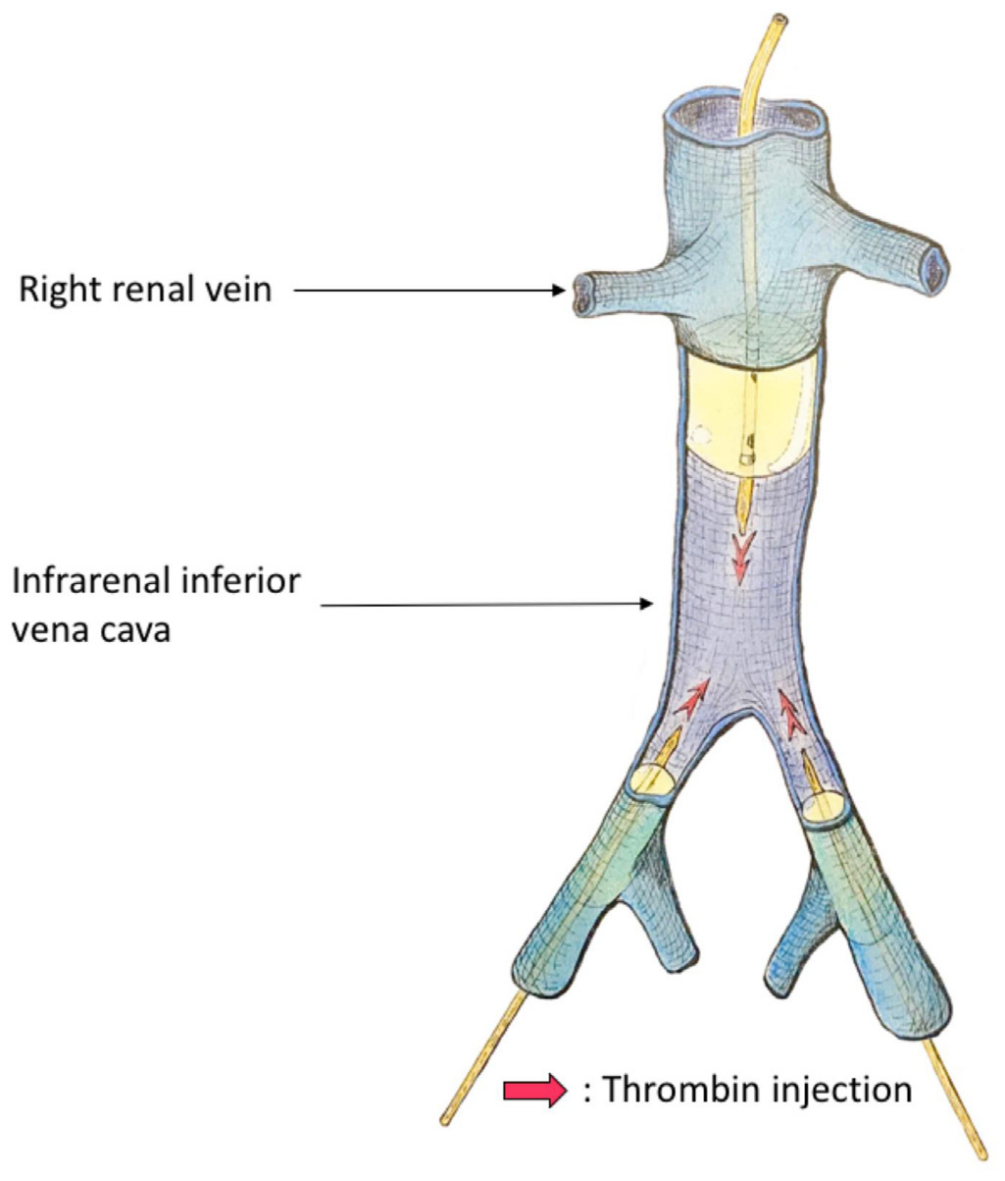
Infrarenal inferior vena cava (*IVC*) thrombosis creation with an endovascular approach.

The experimental protocol consisted of a total endovascular approach. Three large compliant 9F Coda LP balloon catheter (Cook Medical) were inflated to induce venous stasis in the IVC and bilateral common iliac veins. Hypercoagulability was induced by injecting human recombinant thrombin (EVICEL Fibrin Sealant [Human]; Johnson & Johnson) through the balloon catheters, according to the model we previously described^10^ (Fig 2).

After a total of thrombin injection of 10,000 IU and 2.5 hours of waiting time, we gently deflated and retrieved the three balloon catheters. The pigs were then closely monitored daily by the veterinary team.

### Image analysis

At the beginning of the procedure, the pigs first underwent a baseline contrast-enhanced MRI (1.5 Tesla; Aera; Siemens Healthcare GmbH) of the region of interest (ROI), defined as the venous segment between the infrarenal portion of the IVC below the right renal vein and the confluence of the external and internal iliac veins. Next, two post-thrombosis MRI scans were performed: one immediately after creation of central venous thrombosis (MRI_2_) and the other on the day of imaging follow-up and animal euthanasia. After euthanasia, the thrombosed IVC was harvested en bloc and processed for histological examination.

We used gadoterate meglumine (DOTAREM; Guerbet LLC) as the blood pool contrast agent at a concentration of 0.05 mmol/mL. We systematically injected a syringe of 20 mL of gadoterate meglumine through a venous catheter in the animal's ear at the speed of 2 mL/s. Approximately 10 minutes after contrast injection, equilibrium phase, T_1_-weighted, three-dimensional gradient recalled, echo sequence using volumetric interpolated breath-hold examination (VIBE) with a typical flip angle of 10°, echo time of 2.3 ms, repetition time of 4.71 ms, in-plane spatial resolution of 1.0 mm × 1.0 mm, and slice thickness of 1.0 mm was performed (T_1_ VIBE 1 mm). This was used as the reference anatomical sequence.

To assess various MRI parameters of the thrombus, we performed three other MRI sequences.

- T_1_ mapping (two-dimensionally modified look-locker inversion recovery [MOLLI]) with a typical flip angle of 35°, echo time of 1.12 ms, repetition time of 360.56 ms, in-plane spatial resolution of 2.0 mm × 2.0 mm, and slice thickness of 6.0 mm, acquired before (precontrast “native” T_1_ MOLLI) and after (postcontrast T_1_ MOLLI) contrast injection.
- T_2_ mapping with a typical flip angle of 180°, echo time of 60 ms, repetition time of 548 ms, in-plane spatial resolution of 2.0 mm × 2.0 mm, and slide thickness of 6.0 mm, acquired before contrast injection (precontrast “native” T_2_ MOLLI).

In the end, multiparametric MRI consisted of measurements of native T_1_, postcontrast T_1_, and native T_2_.

### MRI feature analysis

**Fig 3:**
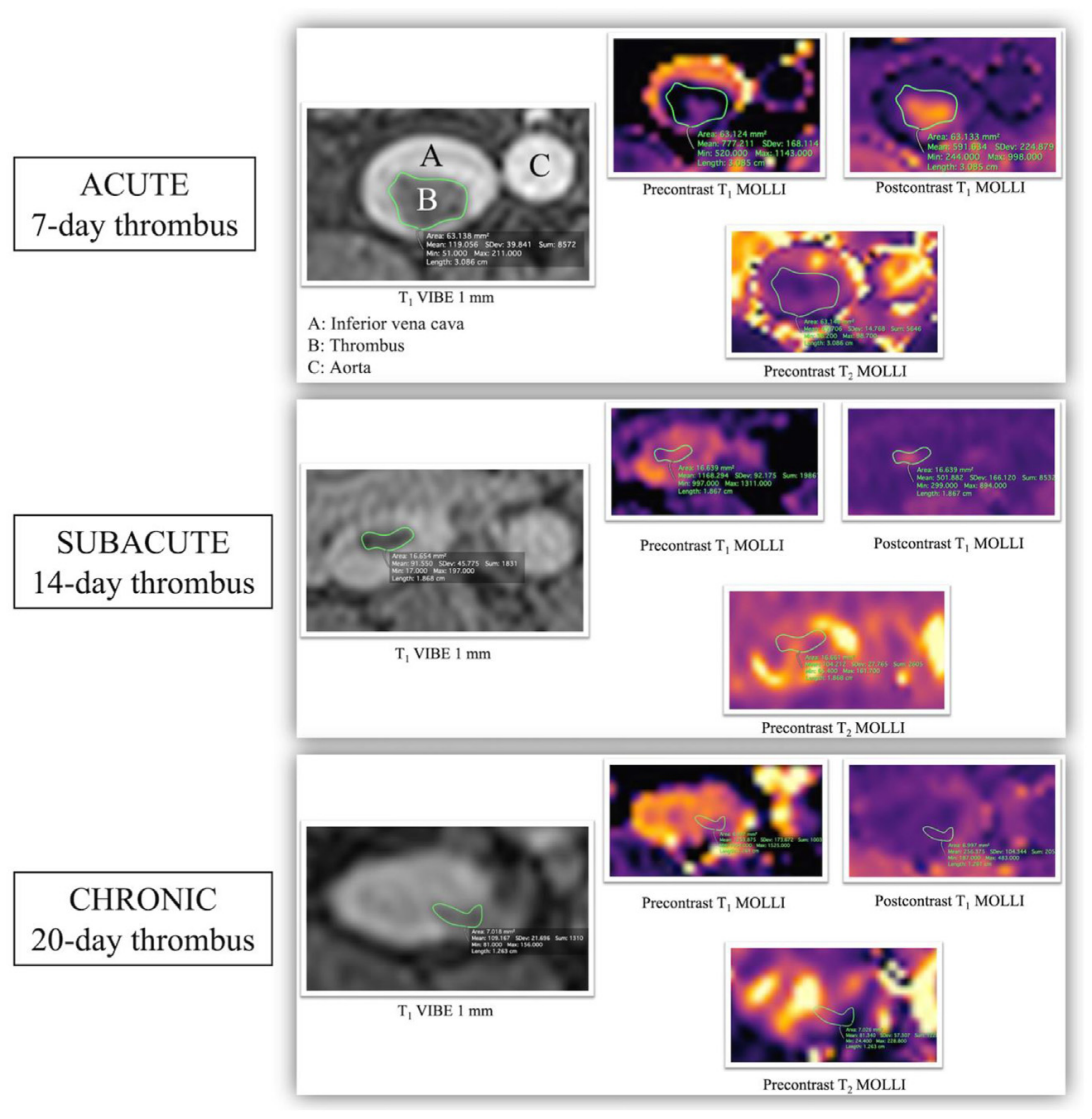
Segmentation of a 7-, 14-, and 20-day inferior vena cava (IVC) thrombus on four different magnetic resonance imaging (MRI) sequences. The anatomical T_1_ volumetric interpolated breath-hold examination (VIBE) 1-mm sequence is used to delineate the exact location of the thrombus, easily seen as a hypointense defect. The segmentation is then propagated to multiparametric MRI maps of precontrast T_1_ and T_2_ and postcontrast T_1_, enabling precise measurement of these parameters. In the acute stage, before contrast injection, the thrombus appears hypointense on T_1_ and T_2_. Contrast injection makes the thrombus hyperintense on T_1_. In the subacute stage, before contrast injection, the thrombus appears isointense on T_1_ and T_2_. Contrast injection makes the thrombus appear hyperintense on T_1_. In the chronic stage, before contrast injection, the thrombus appears isointense on T_1_ and hypointense on T_2_. Contrast injection does not enhance the intensity of the thrombus.

For each pig, we first segmented with open-source OsiriX software (OsiriX DICOM Viewer, version 12.0) the acquisitions of the IVC using the anatomical T_1_ VIBE 1-mm sequence every 10 mm to define a ROI in which the thrombus appeared hypointense and could be delineated and saved. We then searched for the corresponding images for the three multiparametric MRI sequences. The superposition of the ROI previously created on the high-resolution T_1_ VIBE 1-mm sequence on the other sequences made it possible to extract the desired analysis features: T_1_ relaxation times before and after contrast injection and T_2_ relaxation times before contrast injection. Relaxation times are MRI features that reflect the rate of hydrogen nucleus recovery following perturbation using a radiofrequency pulse in the scanner. Relaxation occurs by two independent processes, referred to as longitudinal (T_1_) and transversal (T_2_) relaxation.^11^ These features are usually expressed in milliseconds. This data extraction was performed with OsiriX software, with a mean number of 7.6 ± 3.0 images analyzed for each pig and each sequence (Fig 3).

### Thrombus volume analysis

By precise segmentation using the same software, we performed, using the MRI scans, especially the T_1_ VIBE 1-mm sequence, volumetric reconstructions of the ROI. This allowed us to measure the thrombus volume contained in the ROI in the immediate postoperative period (MRI_2_).

### Statistical analysis

Data are presented as the mean ± standard deviations for continuous variables and as percentages for dichotomous variables, unless otherwise stated. A *P* value < .05 was considered significant. The Kruskal-Wallis test and the Student *t* test were used to compare the MRI features on the different MRI sequences between the three stages of chronicity and the thrombus volumetric reconstructions on MRI_2_. To assess the degree of correlation and agreement between measurements, the parameters were delineated twice by two of us (L.M., K.F.), double-blind, to compute an intraclass correlation coefficient (ICC), calculated by mean squares obtained through analysis of variance.^12^ Statistical analysis was conducted with Stata statistical software (Stata/BE 17.0 for Mac).

## Results

### Thrombus volumetric reconstruction analysis

The total thrombus volume created in the ROI was 17.2 ± 6.3 cm^3^. The comparison of thrombus volume between the animals showed no statistically significant difference (χ^2^ = 13, with 12 degrees of freedom; *P* = .45). Thus, the pigs were comparable in the immediate postoperative period. Moreover, the comparison of thrombus volume between the domestic and Yucatan pigs showed no statistically significant difference, with a mean total thrombus volume in the ROI of 18.8 ± 3.8 cm^3^ and 17.4 ± 5.4 cm^3^, respectively (*P* = .58).

### T_1_ and T_2_ relaxation time analyses

Analysis of T_1_ relaxation times before contrast injection did not show any statistically significant difference between the three groups of chronicity. Analysis of the T_1_ relaxation times after contrast injection showed a statistically significant difference between the three groups (χ^2^ = 7.29, with 2 degrees of freedom; *P* = .026). The AG was significantly different from the CG (*P* = .003), and the SAG was significantly different from the AG (*P* = .027).

There was a statistically significant difference between the three groups regarding the T_2_ relaxation times before contrast injection (χ^2^ = 6.5, with 2 degrees of freedom; *P* = .038). The AG was significantly different from the SAG (*P* = .027), and SAG was significantly different from CG (*P* = .004; Table I).

**Table I:** Variation in T_1_ and T_2_ relaxation times and magnetic resonance imaging (MRI) intensities according to stage of chronicity of inferior vena cava (IVC) thrombosis

Variable	Acute group	Subacute group	Chronic group
Precontrast T_1_ relaxation time, ms	1287.3 ± 401.8 (hypointense T_1_)	1411.1 ± 210.9 (isointense T_1_)	1327.4 ± 130.9 (isointense T_1_)
Postcontrast T_1_ relaxation time, ms	360.2 ± 102.5 (hyperintense T_1_)	354.1 ± 89.7 (hyperintense T_2_)	336.7 ± 55.2 (isointense T_1_)
Precontrast T_2_ relaxation time, ms	160 ± 86.7 (hypointense T_2_)	142.3 ± 55.4 (isointense T_2_)	178.4 ± 11.7 (hypointense T_2_)

The ICCs calculated for the MRI features obtained with OsiriX software are presented in Table II. For all the measures, the ICC varies from 0.88 (95% confidence interval, 0.79-0.93) to 0.98 (95% confidence interval, 0.98-0.99), showing that the quality of the measurement was excellent.^13^

**Table II:** Intraclass correlation coefficients (ICCs) and 95% confidence intervals for magnetic resonance imaging (MRI) features

Variable	ICC	95% CI
Acute group		
Precontrast T_1_ relaxation times	0.93	0.88-0.96
Postcontrast T_1_ relaxation times	0.94	0.90-0.96
Precontrast T_2_ relaxation times	0.93	0.87-0.96
Subacute group		
Precontrast T_1_ relaxation times	0.88	0.79-0.93
Postcontrast T_1_ relaxation times	0.97	0.95-0.98
Precontrast T_2_ relaxation times	0.93	0.88-0.96
Chronic group		
Precontrast T_1_ relaxation times	0.95	0.89-0.97
Postcontrast T_1_ relaxation times	0.95	0.91-0.97
Precontrast T_2_ relaxation times	0.97	0.92-0.98

*CI,* Confidence interval.

## Discussion

This study highlights that multiparametric MRI could be used to date the thrombus according to our protocol, showing MRI parameters characterizing iliocaval venous thrombosis in a swine model at different stages of chronicity. We found that the postcontrast T_1_ relaxation times and precontrast T_2_ relaxation times were significantly different between the three groups of chronicity.

The choice of an appropriate treatment, whether medical or surgical, depends on the date of clinical symptom onset. This paradigm of therapeutic management is based on several unproven hypotheses, including that DVT symptoms appear at the beginning of the DVT process; the progression of DVT is a linear trajectory over time; and the arbitrary choice of a threshold of 14 days.^14^

The 14-day threshold has been demonstrated to be a factor associated with better results for thrombus removal strategies.^4^ However, no consensus has been reached regarding the optimal timing for efficient thrombus removal strategies. Thresholds of both 14 and 21 days after symptom onset have been used in randomized controlled trials.^15^, ^16^, ^17^ The current clinical practice guidelines also do not give a consensual answer. Although the American guidelines indicate a 14-day threshold, the European ones remain imprecise and only recommend “early” thrombus removal strategies.^18^

The need for an accurate, noninvasive diagnostic method of thrombus dating seems obvious. We chose this arbitrary 14-day threshold to distinguish the subacute stage from the chronic stage, with a mean thrombus age of 15.7 ± 2.9 days and 28 ± 5.7 days, respectively. Several noninvasive methods using different modalities have shown their potential in thrombus dating.

Ultrasound-related techniques have been widely investigated for determining venous thrombus age. In particular, elastography has quickly become a method of choice in the attempt to chronologically classify thrombus. It is a noninvasive technique that uses the deformation induced by manually compressing the transducer against the tissue of interest and measuring its displacement.^5^ Such results allow one to classify tissues according to their elasticity. Several preclinical and clinical studies found elastography and strain analysis to be efficient in differentiating acute from chronic thrombi; however, these techniques are still unable to prospectively estimate the efficiency of thrombus removal or lysis.^19^^,^^20^

A few studies showed the ability of specific radiolabels to assess thrombus age. Technetium-99m-apticide showed good results in differentiating between acute and chronic thrombi and was used to radiolabel activated platelets in patients with DVT.^21^

MRI is an accessible and noninvasive technique allowing a morphological vascular analysis and a precise study of thrombus content characterized by the variation of different MRI features, as reported in the present study. In 2014, Arnoldussen et al^22^ reported the change of thrombus particularities over time on MRI, allowing for qualification of thrombosis as acute and subacute with morphometric parameters. We have furthered their findings by presenting the variation of objective MRI parameters to best characterize these three stages of chronicity. To the best of our knowledge, this has not been previously reported.

MRI became a keystone for noninvasive cardiac functional explorations, complementing clinical results and echocardiographic findings. Its spatial resolution makes it possible to appreciate the different components of a structure due to the variation in image intensities and T_1_ relaxation times, which are specific to each tissue. Multiparametric MRI is an original quantitative approach with maps of different MRI-specific parameters demonstrated to be directly related to tissue composition. Therefore, native T_1_, native T_2_, and postcontrast T_1_ values are directly linked to tissue specificity and could serve as “fingerprinting” for deeper tissue characterization.^23^

The key to understanding these MRI signal variations could be to establish correlations between the MRI results and those obtained by histological analysis. Our previous study presenting the animal model included a histological analysis obtained after animal euthanasia at different time points.^10^ The composition and histological findings of the thrombus contribute to the different mean T_1_ and T_2_ relaxation times obtained in this study. However, these cannot be analyzed quantitatively on MRI without the use of specific, targeted contrast agents, such as those available for fibrin and collagen.^24^^,^^25^

MRI avoids radiation and brings more precise depiction of thrombus than computed tomography, including the proximal and distal extent, age, and any IVC abnormality,^26^ with the advantage of highlighting many causes of secondary IVC thrombosis.^1^ Disadvantages include accessibility and cost, with a remaining risk of nephrogenic systemic fibrosis in the case of injection of gadolinium-based contrast agents in patients with renal dysfunction.^27^^,^^28^ However, native T_2_ mapping is obtained without gadolinium injection and could be used to help date thrombus without using contrast.

Although our study found good ICCs, testifying to the excellent quality of the measurements, this data extraction remains a manual operation, which can be a source of errors. If radiological results could be segmented by a computer-aided approach to delineate the ROI and provide quantitative graphic variables relevant to the diagnosis, appropriate graphic features could be selected for the diagnosis of specific lesions, minimizing inter-radiologist variations and providing objective and quantitative radiological data.^29^

We chose a large animal model, because these animals have a coagulation profile relatively close to that of humans.^30^ The vascular anatomy of the pig is also to some extent comparable to that of humans and allows for the use of endovascular devices used in routine practice in the operating room. Finally, it is an easily usable and accessible model. Unlike most currently described large animal models of central venous thrombosis, our model allows us to remain totally endoluminal during the creation of the thrombosis and, thus, respect the venous anatomy. By ensuring that we remain completely endovascular, we prevent direct injuries to the venous segment of interest and allow for a better aperception of the customary venous pathophysiology. This allows us to assess and understand the short- and long-term outcomes of DVT in actual and representative diseased veins, which has never been the case to date in a large animal model.

### Study limitations

The first limitation is the small number of animals. Second, as in humans, pigs have interanimal variability that we could not control, although we selected animals of comparable size and morphology. We used two porcine breeds, domestic swine and Yucatan, due to veterinary recommendations. The basis for this suggestion was that experience supported the idea that the Yucatan pig would be more robust during and after the procedure. Third, only one type of MRI scanner was used, and generalization to other magnet strengths such as 3T scanners or other mapping sequences cannot be guaranteed. Because the research must be translational, this study must be continued using these specific MRI sequences in patients with central DVT of different ages to investigate whether extrapolation of our results to humans is possible.

## Conclusions

This study allowed us to highlight multiparametric MRI characteristics in a swine model, including thrombus volumes and T_1_ and T_2_ relaxation times, that could have the potential to significantly differentiate subacute and chronic stages from an acute stage of central DVT in humans. Further clinical studies in humans are warranted.

In addition to providing a better understanding of venous thrombosis remodeling over time, MRI has the potential to be a tool that could allow us to characterize the content of venous thrombosis over time, allowing for a refined analysis of the local evolution of venous thrombosis. Being able to determine these features noninvasively is critical for vascular surgeons when it comes to choosing between fibrinolytic therapy or surgical management. Currently, algorithms in the management of venous thrombosis are mainly based on expert opinions rather than evidence, and these data have the prospect of paving the road toward evidence-based guidelines.

## Author contributions

Conception and design: LM, AS, DS, KG, NC, JB

Analysis and interpretation: LM, KF, JB

Data collection: LM, AS, PC, KA, JB

Writing the article: LM, AS, NC, JB

Critical revision of the article: LM, AS, PC, KF, KA, DS, KG, NC, JB

Final approval of the article: LM, AS, PC, KF, KA, DS, KG, NC, JB

Statistical analysis: LM

Obtained funding: AS, JB

Overall responsibility: LM

## Disclosures

None.
